# Transcript copy number estimation using a mouse whole-genome oligonucleotide microarray

**DOI:** 10.1186/gb-2005-6-7-r61

**Published:** 2005-06-30

**Authors:** Mark G Carter, Alexei A Sharov, Vincent VanBuren, Dawood B Dudekula, Condie E Carmack, Charlie Nelson, Minoru SH Ko

**Affiliations:** 1 Developmental Genomics and Aging Section, Laboratory of Genetics, National Institute on Aging, National Institutes of Health, 333 Cassell Drive, Baltimore, MD 21224, USA; 2 Agilent Technologies, Deer Creek Rd, Palo Alto, CA 94304, USA

## Abstract

The ability to quantitatively measure the expression of all genes in a given tissue or cell with a single assay is an exciting promise of gene-expression profiling technology. An *in situ*-synthesized 60-mer oligonucleotide microarray designed to detect transcripts from all mouse genes was validated, as well as a set of exogenous RNA controls derived from the yeast genome (made freely available without restriction), which allow quantitative estimation of absolute endogenous transcript abundance.

## Abstract 2

An *in-situ*-synthesized 60-mer oligonucleotide microarray designed to detect transcripts from all mouse genes is presented. Exogenous RNA controls derived from yeast allow quantitative estimation of absolute endogenous transcript abundance

## Background

One of the most tantalizing promises of gene-expression profiling technology has been to develop assays that measure expression of all genes in a given species [1]. This is especially important for the mouse, which is a standard model for various human diseases. The early and rapid development of murine bioinformatics resources such as the draft genome assembly [2] and numerous expressed sequence tag (EST) projects have bolstered the feasibility of developing such microarray platforms for the mouse. However, because it has been difficult to identify all murine genes and correctly group genomic and expressed sequences into genes and transcripts, microarray platforms intended to cover all mouse genes are only now being made widely available, long after the draft assembly was released.

Relatively recent microarray technologies, which require sequence information instead of clones as input, allow investigators to design microarray platforms to detect genes without having to obtain clones, including genes which have yet to be cloned or confirmed as an expressed transcript [3]. Platforms that utilize long oligonucleotides give high sensitivity, with the potential for transcript specificity sufficient to distinguish transcripts from the same locus or closely related gene-family members [4,5].

While microarray-based methods can provide very accurate relative (ratio-based) expression measurements, they usually do not provide absolute expression measurements (that is, transcript copy number). One notable exception described in the literature does provide absolute expression measurements in yeast, but not as copy numbers [6]. That method relies on labeled oligonucleotides complementary to common sequence in each cDNA probe, which are hybridized against each slide as the reference target. In the case of long-oligonucleotide-based microarrays, there is no sequence common to all probes, so such a strategy is not feasible. An appropriate approach for such microarray platforms is to monitor the hybridization behavior of a few spiked-in RNA controls with sequence derived from yeast or other genomes. Control transcript probe intensity data can be used to create a generalized dose-signal model and applied to endogenous transcript intensity data to give transcript abundance estimates. Not only would such absolute expression measurements from microarrays help determine what level of sensitivity is required for downstream validation methods, but they would also allow direct comparison of expression data generated using different methods, as well as a valuable mechanism to compare performance between slides, platforms, or experiments [7]. Most importantly, global absolute expression measurements can be used to more fully describe a given transcriptome, perhaps identifying mRNAs present at less than one copy per cell as candidates for heterogeneous or cell-type-specific expression, or subdividing groups of genes in Gene Ontology (GO) nodes [8] based on transcript abundance.

The work described here is focused on two goals, aimed at facilitating standardization and comparison among mouse microarray studies: first, to create a long-oligonucleotide-based microarray platform covering all identified mouse genes, which can be made widely available; and second, to develop exogenous RNA controls which will allow quantitative estimation of absolute endogenous transcript abundance. The microarray will be made available to the community through Agilent Technologies and exogenous control plasmid vectors will be available upon request from the authors and the American Type Culture Collection (ATCC) (ATCC MBA-201 to -207) without restriction, to be used with the design presented here or incorporated into any non-yeast microarray platform.

## Results and discussion

The development of a mouse whole-genome microarray in our laboratory has been an ongoing effort, and each new design has been derived in part from its predecessor (see Additional data files 1 and 2 and Materials and methods for details) [9]. Development of the National Institute on Aging (NIA) Mouse Gene Index [10] facilitated more complete, less redundant microarray design than EST clustering alone for the following reasons. First, clustering was mapped to the genome assembly, improving consolidation of transcriptional units. Second, transcript selection is no longer restricted to library contents, allowing genes absent from NIA cDNA clone collections [11] to be included from other public sequence collections. Finally, all potential splice variants were solved from EST alignments with genomic sequence, so that probes can be designed to common regions in a transcript family, minimizing the effect of differential splicing. Therefore the index has been the basis of gene/transcript identification and sequence selection for all oligonucleotide array designs subsequent to the NIA Mouse 22K Microarray v1.1. During the preparation of this paper, assembly of a long-oligonucleotide microarray platform with full coverage of the mouse genome was reported by Zhang *et al*. [12] using a sequence selection protocol that incorporated all National Center for Biotechnology Information (NCBI) RefSeq entries, including all mRNA transcripts based solely on prediction algorithms, without experimental evidence of expression (XM sequences). In contrast, our protocol included only a minority of the XM sequences (only those annotated as an identified gene).

**Figure 1:**
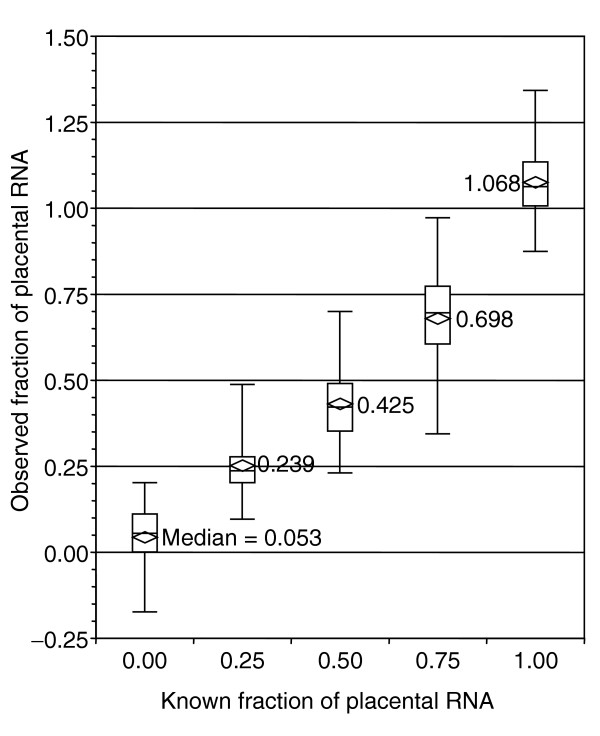
60-mer oligonucleotide probe linearity testing. To test the performance of 21,044 60-mer oligonucleotide probes, E12.5 embryo RNA and placenta RNA were combined to form five pairs of duplicate samples containing from 0 to 100% placental RNA. Box-plot distribution data for each placental RNA input level is shown above, with median values labeled. The boxes show the 25-75 percentile range, with the mean and median indicated by the central straight line and diamond, respectively. Upper and lower bars show the 2.5 to 97.5 percentile range. Observed fraction medians are within 0.075 of input values, and 95% of values are within 0.405 of input values.

As our oligonucleotide probe design and selection process differed slightly from protocols previously used with ink-jet microarrays, we first established that our oligonucleotide probes perform as well as or better than those designed with standard protocols [5,9,13]. To assess the overall performance of the oligonucleotide probes, we carried out a mixing experiment, combining total RNA from E12.5 mouse embryos and placentas to produce a range of gene-expression ratios for each transcript, using a preliminary microarray design (NIA Mouse 22K Microarray v2.0, see Additional data files 1 and 2 for details). In a comparison of E12.5 mouse embryo and placental RNA, statistically significant differential expression was detected for 8,461 of the test array's 21,044 oligonucleotide probes. These differential targets were then examined in the mixtures to calculate observed placental RNA fractions. Figure 1 shows that the distributions of the observed placental RNA fractions at each input level were closely matched with the input placental RNA fractions (median observed fraction = input fraction ± 0.075), and the boundaries of 95% confidence regions were 0.121 to 0.405 from the median. These distributions were consistent with, although narrower than, those seen in a similar study [13] using standard oligonucleotide design procedures, suggesting that our design protocol produces comparable results. More importantly, these data suggest that the oligonucleotide probes are capable of highly quantitative, proportional measurements of transcript abundance, a property required for transcript abundance estimation.

**Figure 2:**
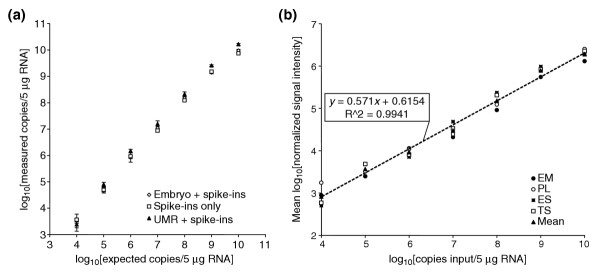
Relating yeast spike-in RNA control copy number to qPCR measurements and microarray signal intensity. **(a) **To verify abundances of yeast sequence RNA transcripts in a control mixture, cDNA was transcribed from the control mixture alone (open boxes), as well as E12.5 whole-mouse embryo total RNA (open diamonds) and Universal Mouse RNA (filled triangles) with added spike-in control mixture. The cDNA was used as template for real-time PCR quantitation of each yeast sequence RNA, using a separately prepared standard of cDNA transcribed from the yeast sequences. Expected and measured copy numbers are closely matched (*r*^2 ^≥ 0.99), with maximum measured/observed ratios of 1.5, 1.5, and 2.6, respectively. **(b) **Expression profiles were generated for triplicate total RNA samples from E12.5 embryo (filled circles), E12.5 placenta (open circles), ES cells (filled boxes), and TS cells (open boxes) with yeast sequence control transcripts spiked-in prior to target labeling. For the seven control transcripts, mean log_10_[intensity] is shown for each tissue type, as well as the mean across all samples (filled triangles), and these data were used to perform linear regression analysis and relate signal intensity to transcript copy number, allowing abundance estimation for endogenous transcripts. The regression line for the average of all tissues (dashed line) and its equation is shown. Intensity-copy number correlations for individual tissues were very strong, with *r*^2 ^values of 0.98 - 0.99.

Exogenous RNA control transcripts were developed from *Saccharomyces cerevisiae *intronic and intergenic sequences [14,15]. A total of 11 candidate sequences were cloned and tested against multiple oligonucleotide probes in preliminary microarray hybridizations (data not shown). After assessing which target/probe pairs produced the best dynamic responses to abundance with the lowest noise, seven control transcripts and corresponding oligonucleotide probes (Tables 1 and 2) were selected for use in the control set. As a result, the NIA Mouse 44K Microarray v2.0 contains all 63 oligonucleotide probes considered as controls, while version 2.1, the final version which will be made available to the community, contains only the seven selected for use, spotted ten times each at different locations on the slide. Loading of each control transcript into total RNA was confirmed as accurate within 2.6-fold by quantitative real-time RT-PCR (qPCR) (Figure 2a), with a very tight correlation (r^2 ^≥ 0.99) between expected and measured values over seven orders of magnitude.

**Figure 3:**
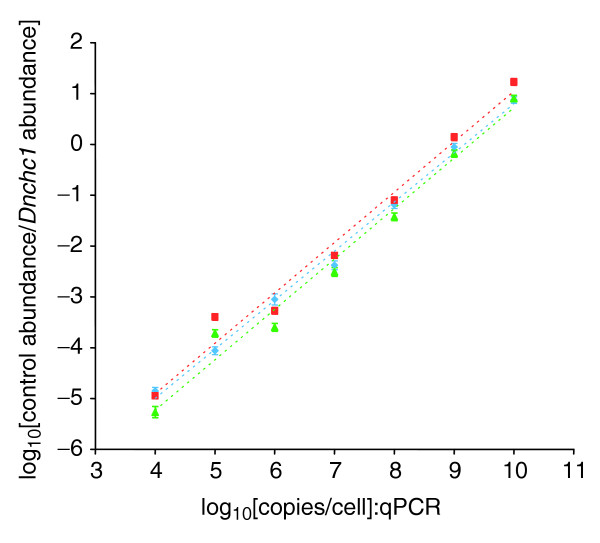
Exogenous control and endogenous transcript amplification rates are closely matched over seven orders of magnitude. Transcript abundance of each spike-in control transcript was measured by qPCR before and after linear amplification labeling, and compared to amounts of the exogenous transcript *Dnchc1*. After amplification, individual ratios of each control transcript to the endogenous transcript were within 3.5-fold (average = 1.98-fold) of those prior to amplification. Blue diamonds = log_10_[ratio mean control/*Dnchc1 *transcripts] of three E12.5 embryo and three E12.5 placenta samples before amplification. Red boxes, green triangles = log_10_[ratio mean control/*Dnchc1 *transcripts] for the same samples after amplification, using yield versus input (red boxes) or the increase in *Dnchc1 *transcripts as measured by qPCR (green triangles) to calculate the fraction of the original sample represented by each qPCR well.

One basic assumption made in our experimental design is that amplification efficiencies are approximately equal between endogenous mouse transcripts and exogenous yeast control transcripts. To test this, transcript abundances were determined by qPCR for cDNA pools synthesized from total RNA with spike-in controls added, as well as labeled cRNA target mixtures amplified from the same total RNA/spike-in control mixtures, and transcript abundances were determined by qPCR. After linear amplification, individual ratios of each control transcript to the endogenous transcript *Dnchc1 *(Table 3) were within 3.5-fold (average = 1.98-fold) of those prior to amplification (Figure 3), and the slopes of regression lines for pre- and post-amplification datasets were 0.967 and 0.992, respectively. Results were consistent whether using amplification yield versus input or the increase in *Dnchc1 *transcripts as measured by qPCR to calculate the fold amplification and fraction of the original sample represented by each qPCR well. The stability of the relationship holds over seven orders of magnitude, suggesting that amplification of transcripts during cRNA microarray target synthesis is not a source of significant bias. In previous attempts using control transcripts with short (20-40 nucleotides) vector-derived poly(A) tails, exogenous controls amplified one or two orders of magnitude less efficiently than endogenous messages (data not shown), indicating that sufficient polyadenylation of controls is critical for efficient amplification.

Microarray expression profiles were generated for three distinct samples each of total RNA from E12.5 whole embryos (EM), E12.5 placenta (PL), R1 embryonic stem cells (ES), and GFP-Exe trophoblast stem cells (TS) [16]. For each microarray, linear regression analysis on mean normalized log_10_[intensity] values for seven yeast spike-in control probes was used to define a standard curve relating signal intensity to copy number (Figure 2b) for estimation of endogenous transcript abundances. Correlations were very strong between log_10_[intensity] and log_10_[input copy number], with r^2 ^≥ 0.95.

**Figure 4:**
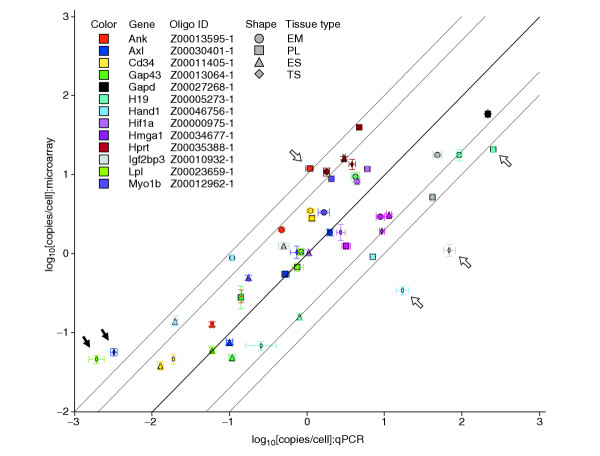
Validation of transcript abundance estimation for endogenous transcripts. qPCR primer sets were designed for selected genes so that amplicons were upstream of 60-mer oligonucleotide probes when possible, or less than 650 bp downstream, and copy number was estimated using serial dilutions of RNA, *in vitro *transcribed from mouse cDNAs, at known copy numbers as standards. Error bars represent one standard deviation across three replicate samples for each tissue. Dotted diagonal lines represent five- and tenfold differences between the two datasets. Each gene's official symbol, along with the unique identifier for the 60-mer oligonucleotide probe it was measured with, are listed in the key. Data was normalized to *Gapd *expression for both methods. EM = E12.5 embryo, PL = E12.5 placenta, ES = embryonic stem cells, TS = trophoblast stem cells.

To test the accuracy of estimating transcript abundance in this way, we compared the results with qPCR measurements for a panel of 13 endogenous transcripts (Figure 4). Most (36 of 52, or 69.2%) of the microarray-based transcript copy-number estimates for a panel of 13 endogenous genes were within fivefold of qPCR measurements. Furthermore, trending for each transcript across the four tissue types was consistent between the two methods for all ten non-housekeeping genes showing differential expression.

Many factors are likely to affect the accuracy of transcript abundance estimates. Measurements at or near the microarray's detection limit, but still above that of qPCR assays (Figure 4, *Lpl *and *Axl *in TS, filled arrows), tend to overestimate transcript abundance, and these data suggest that the lower limit of microarray-based transcript abundance measurement is approximately 0.05 to 0.06 copies per cell in this experiment. Differential transcript splicing can also have an effect: note that for *Ank*, *H19*, *Hand1*, and *Igf2bp3 *(Figure 4, open arrows), only one tissue out of four shows greater than a tenfold discrepancy, whereas the other measurement pairs are more closely matched. Given the preceding discussion, we present this method as a way to estimate transcript abundances for groups of genes. Accuracy of the estimates for each gene/probe may be further improved in the future by studying the effects of various probe-selection parameters on measured fluorescence intensity.

**Figure 5:**
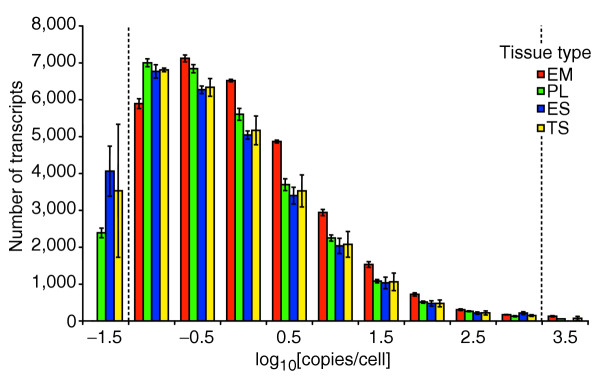
Distribution of mouse transcript abundances in E12.5 embryo and placenta, and cultured ES and TS cells. Transcript abundances are expressed as log_10_[copies per cell], varying over six orders of magnitude. The distributions are highly similar, despite the significant differences between the four tissues (for example, monolayer culture versus tissue, placenta versus embryo), suggesting that such distributions are not heavily skewed according to tissue structure or function. The percentage of transcripts present at less than one copy per cell ranged from 40.1 to 48.2% in the four tissues. Bins were centered on indicated values, and the dotted lines indicate values corresponding to mean upper and lower signal intensity reliability limits of one copy per 26 cells to 2,188 copies per cell. For definitions of tissue type see Figure 4 legend.

Using conservative estimates of the total RNA content recovered from mammalian cells (2.0-3.0 pg/cell in this case, see Materials and methods), transcript abundances were expressed on a copies-per-cell basis (Figure 5). The analysis revealed two striking properties of these transcript-abundance distributions. First, mRNA populations in mammalian tissues are highly complex, which is consistent with previous observations [17,18]. Many transcripts were measured at less than one copy per cell in each tissue (EM = 40.1 ± 0.6%, PL = 46.9 ± 1.3%, ES = 48.2 ± 1.9%, TS = 47.4 ± 3.4%) (Figure 5). A log_10_[intensity] value of 2.5 was used as a lower cutoff, which corresponds to about one copy in 26 cells, so it appears that measured values from 0.038 to one copy per cell represent transcripts present at very low measurable copy numbers, rather than nonexpressed transcripts. Indeed, quantitative RT-PCR studies in yeast have shown that many genes, particularly transcription factors, are expressed at less than one copy per cell [19]. Furthermore, our estimates of numbers of expressed genes/transcripts and mRNA message content per cell (519,688 to 851,087 mRNAs per cell, 8,357 to 12,739 transcripts, expressed from 8,101 to 11,360 genes, Table 4) compare well with previous estimates ranging from 200,000 to 600,000 mRNAs per cell [20,21], consisting of 11,500 to 15,000 diverse mRNA species [18,20], transcribed from as many or more genes up to 17,000 [18,20,22]. Second, a majority of transcripts expressed in one tissue or cell type are commonly expressed in other diverse cell and tissue types. The number of expressed genes in each tissue was estimated by counting the number of microarray features measuring absolute expression of at least one copy per cell, and converting this set of microarray probes to U-clusters (loci) and transcripts via the NIA Mouse Gene Index (Table 4). Examination of the overlap between each cell type's roster of expressed genes and transcripts reveals that the majority are expressed in common (Tables 4 and 5), as suggested by previous assessments of mRNA complexity [18,20,22]. For example, 93% of expressed placental transcripts are also expressed in embryo, and this group represents 72% of the expressed transcripts in embryo (Table 5). The same relationship holds true for pairings of cultured cells with embryo, with 95% of expressed transcripts in cultured cells also found in embryo, covering 69% of embryonic transcripts.

When comparing frequency distributions for complex, *in vivo *samples and less complex *in vitro *cultured cells, we might expect to see large differences, particularly in the case of genes expressed at less than one copy per cell. Transcripts present at less than one copy per cell cannot be present in every cell, and therefore must be expressed heterogeneously. As might be expected, whole embryos had the most distinctive frequency distribution of the four samples examined: embryos had significantly fewer transcripts in the range log_10_[copies per cell] = -1.0 (0.1 copies per cell), but significantly more in the 0-2 (1 to 100 copies per cell) range. This difference, combined with the higher estimate of total transcripts per cell for whole embryos (Table 4), may reflect the activation, within the context of the very high transcriptional activity present in developing embryos, of many developmental pathways that are normally inactive or minimally active.

In contrast, the high degree of similarity between the frequency distributions for placenta, ES, and TS cells (Figure 5) suggests that levels of expression heterogeneity can be similar for complex tissues and cultured cells. In fact, there is evidence in ES cells that gene expression within a culture is not as uniform as previously supposed, and even key differentiation markers such as *Oct4 *and *cKit *are expressed in cellular subpopulations within cultures [23]. Taken together, these observations suggest that cultured ES and TS cells, although clonally isolated, are quite heterogeneous in terms of their gene-expression patterns, with a transcriptional complexity similar to that of E12.5 placenta. Further study, perhaps using *in situ *hybridization or single-cell RT-PCR methods, will be required to address this issue, but it does beg the question of whether or not this heterogeneity is common to all cultured cells, or a feature specific to pluripotent stem cells.

## Conclusion

Here we present an oligonucleotide microarray for gene-expression profiling with representation of the entire mouse genome, according to the NIA Mouse Gene Index version 2.0 [24]. An integral feature of this new whole-genome microarray design is a set of probes detecting yeast spike-in control transcripts, which will be available to the community without restriction. Using qPCR, we have shown that this control system allows the reproducible estimation of absolute transcript levels. A valuable tool for the mammalian functional genomics community, this system is a step towards standardization of microarray results by using exogenous RNA control systems that are compatible with multiple microarray platforms and model organisms.

## Materials and methods

### Microarray design: target sequence selection

The NIA Mouse 44K Microarray v2.0 (Whole Genome 60-mer Oligo) design was based on the NIA Mouse Gene Index v2.0 [24]. Like the first version of the NIA Mouse Gene Index [10], it combines data from multiple transcript databases (RefSeq, Ensembl, Riken, GenBank, and NIA) to construct gene/transcript models which represent all possible transcripts. Briefly, 249,200 ESTs developed at NIA were clustered using clustering tools from The Institute for Genome Reserach (TIGR) [25], generating 58,713 consensus and singleton sequences which were then combined with the other datasets. The major difference in version 2 from version 1 is the use of a clustering method based on genome alignments rather than sequence homology between NIA EST clusters and public sequences. Individual sequences were aligned to the mouse genome [2] using BLAT [26], then clustered by an algorithm similar to the one described by Eyras *et al*. [27], to be published elsewhere. Our assembly included 30,796 primary genes and 1,318 gene copies or pseudogenes, as well as 28,928 clusters that did not match our criteria for high-confidence genes (open reading frame (ORF) of more than 100 amino acids or multiple exons). There were 65,477 transcripts associated with primary genes. Because transcripts were built from sequence alignments to the mouse genome, they match published genomic sequences [2] (February 2003 edition) exactly.

### Microarray design: oligonucleotide probe design and selection

In designing a mouse whole-genome microarray, we began by examining existing designs - the NIA Mouse 22K Microarray v1.1 (Development 60-mer Oligo) [9], which became commercially available from Agilent as the Agilent Mouse (Development) Oligonucleotide Microarray (see Additional data files 1 and 2), and the National Institute of Environmental Health Sciences (NIEHS) Toxicogenomics Consortium mouse array (Agilent Mouse Microarray). Criteria for selecting previously designed probes included a good match to the target gene's major transcript with the longest ORF, minimum predicted cross-reactivity with other expressed sequences, and nonredundancy. Although a perfect match of all 60 base-pairs (bp) of the oligonucleotide was preferred, we also accepted up to two mismatches to the genome if the oligonucleotide matched perfectly to the RefSeq sequence, and oligonucleotide sequences that did not match 100% to the RefSeq entry were corrected. An oligonucleotide was considered cross-reactive if its last 43 bp (solution end) matched to a non-target gene with less than five mismatches. Deletion placement studies using *in-situ *synthesized 60-mer oligonucleotide probes suggest that the 17 bp at the support surface have a negligible effect on hybridization intensity [5]; thus only the external 43 bp were considered important. While the cross-reactivity criterion is easily satisfied for unique genes with low similarity to other genes, many gene families had high sequence similarity between member transcripts, and it was impossible to find regions with low predicted cross-reactivity. In this case we considered the whole gene family as a target; then the oligonucleotide was considered cross-reactive only if it matched to genes outside the family. Gene families were assembled using a 30% transcript length alignment as a threshold of similarity; alignments for each pair of transcripts were generated using BLAT [26]. According to the nonredundancy criterion, we left only one oligonucleotide that matched to each gene or gene family, and when probes from both the NIA Mouse 22K v1.1 and NIEHS Toxicogenomics arrays matched well to the same gene, preference was given to the NIA oligonucleotide.

After filtering with the above criteria, we obtained 6,563 probes from the NIA Mouse 22K Microarray v1.1 and 9,551 probes from the NIEHS Toxicogenomics array. Among these oligonucleotides, 3,327 did not match the target gene's major transcript with the longest ORF, so we generated an additional 3,327 probes for major transcripts of the same genes. Then we generated 22,850 probes for the best transcripts of primary genes in the gene index that were not represented in the NIA Mouse 22K Microarray v1.1 (Development 60-mer Oligo) and NIEHS Toxicogenomics arrays, for a total of 42,291 non-control oligonucleotide probes (see Additional data file 2). For each transcript we generated ten probes using ArrayOligoSelector [28], then selected the best oligonucleotide on the basis of minimum predicted cross-reactivity, proximity to the 3' end, and degree of matching to RefSeq or GenBank sequences. The latter criterion was important only in cases of mismatches between genomic sequence and RefSeq or GenBank.

All microarray data described in this report were generated using the NIA Mouse 44K Microarray v2.1 (Whole Genome 60-mer Oligo) and NIA Mouse 22K Microarray v2.0 (Development 60-mer Oligo). We have slightly modified the probe content of the NIA Mouse 44K v2.0 array by including Agilent's standard QC probe set, removing candidate spike-in control probes which were not used, and including additional probes for known genes that have existing probes with poor performance or ambiguous targeting. The updated version (NIA Mouse 44K Microarray v2.1 (Whole Genome 60-mer Oligo) will be made available to the community (see Additional data file 1).

### Yeast spike-in controls

Yeast (*S. cerevisiae*) sequences were selected from public repositories [14,15] to produce exogenous RNA control transcripts, commonly referred to as 'spike-in' controls. Fourteen candidates (ten intergenic and four intronic) were selected on the basis of sequence length and the absence of restriction endonuclease cleavage sites important for our cloning strategy. Sequences with significant matches to transcripts in the NIA mouse Gene Index v2.0 [10] were discarded, and ten of the 14 remaining candidates were successfully cloned from genomic DNA, with one sequence divided into two clones for a total of 11 potential controls. Yeast sequences were amplified with added 5' *Sal*I and 3' *Xba*I sites from *S. cerevisiae *genomic DNA (ATCC 2601D) using Sigma RedTaq, and cloned directly into pCR4-TOPO (Invitrogen). TA-TOPO clones were verified by sequencing on an Applied Biosystems 3100 capillary DNA sequencer, and inserts were directionally subcloned into pSP64 Poly(A) (Promega Catalog number P1241) using the introduced *Sal*I and *Xba*I sites. A total of 63 60-mer oligonucleotide 'sense-strand' probes were selected for the 14 candidate sequences using both ArrayOligoSelector software [28] and arbitrary manual selection. Oligonucleotide probes were compared to NIA Gene Index transcripts, and no significant matches were found. Control probes were spotted ten times each in various locations throughout the slides.

Spike-in RNA was transcribed, polyadenylated, and purified using Ambion mMessage mMachine, poly(A) tailing, and MegaClear kits, then sized and quantitated by RNA 6000 Nano assay on an Agilent Bioanalyzer 2100. Spike-in RNAs were pooled to create tenfold concentration differences, from 10^4 ^to 10^10 ^copies per microliter (Table 1). Before preparation of microarray targets, 1 μl of this control transcript mixture was added to 5-μg aliquots of each total RNA sample, including the reference RNA. A separate pool with all yeast control transcripts present at the same copy number was added to reference RNA and converted to cDNA for use as a standard in qPCR assays.

### RNA collection/preparation

Total RNA was prepared using TriZol reagent (Invitrogen) from E12.5 C57BL/6J embryos, pooled by litter, and corresponding E12.5 C57BL/6J placenta pools [9]. Total RNA was also prepared from R1 ES cells passaged briefly on gelatin to remove feeder cells, and GFP-Exe TS cells grown on plastic in conditioned medium as previously described [16]. Total RNA quantity and quality were assessed by RNA 6000 Nano assay. For oligonucleotide signal linearity testing, E12.5 embryo and placenta total RNA were pooled, based on this quantitation, to produce duplicate samples with 0, 25, 50, 75, and 100% placental RNA content.

### cRNA target labeling

Fluorescently labeled microarray targets were prepared from 2.5 μg aliquots of total RNA samples with yeast sequence control mixtures added as described above, using a Low RNA Input Fluorescent Linear Amplification Kit (Agilent). A reference target (Cy5-CTP-labeled) was produced from Stratagene Universal Mouse Reference RNA, and all other targets were labeled with Cy3-CTP. Targets were purified using an RNeasy Mini Kit (Qiagen) as directed by Agilent's clean-up protocol, and quantitated on a NanoDrop scanning spectrophotometer (NanoDrop Technologies).

### Microarray hybridization

All hybridizations compared one Cy3-CTP-labeled experimental target to the single Cy5-CTP-labeled reference target. Microarrays were hybridized and washed according to Agilent protocol G4140-90030 (Agilent 60-mer oligo microarray processing protocol - SSC Wash, v1.0)*. *Slides were scanned on an Agilent DNA Microarray Scanner, using standard settings, including automatic PMT adjustment.

### Real-time quantitative RT-PCR

Primer sets were designed and tested for SYBR Green chemistry using an established in-house protocol [9]. Total RNA was used to prepare cDNA as described previously [9]. Because the microarray targets were oligo(dT) primed, all cDNA synthesis reactions were oligo(dT) primed as well, and qPCR primer sets were designed so that amplicons were upstream of 60-mer oligonucleotide probes when possible, or less than 650 bp downstream. These steps were taken to minimize the effects of 3' end-labeling bias from microarray target synthesis. Yeast spike-in standard curve cDNA was prepared by mixing equal copy numbers of each synthetic yeast RNA with Mouse Universal Reference total RNA, followed by cDNA synthesis. A standard for copy-number measurement of endogenous mouse genes was prepared by transcribing cDNA clones and adding these transcripts in equal numbers to yeast total RNA, followed by cDNA synthesis. A BioMek 2000 liquid-handling system (Beckman) was used to aliquot cDNA into 96- and 384-well plates, then assemble and aliquot PCR master mix into 20-25 μl reactions. Plates were run on ABI 7700 or ABI 7900 HT Sequence Detection Systems using the default cycling program, and data was processed using SDS 1.9 or SDS 2.2 software (Applied Biosystems) and Microsoft Excel.

### Data analysis

Microarray images were processed with Agilent Feature Extractor A.7.5.1 software to generate normalized, background-subtracted feature intensities. Dye normalization was performed by applying a LOWESS algorithm to all significant, non-control and non-outlier features. Analysis of variance (ANOVA) and replicate averaging was performed as previously described [9] using NIA Array Analysis Tool software [29], which normalizes each probe according to reference RNA signals.

For each probe identified as differentially expressed in mixing experiments (false discovery rate < 0.05) [9], linear regressions of ratios against pure placental RNA across the five levels of placental RNA content were calculated, and observed ratios were back-calculated for population analysis as



where *P*_*oi *_is the observed fraction placental RNA content calculated from a given probe *i*, *I*_*pi *_and *I*_100*i *_are the normalized log_10_[intensity] values for the probe *i *at placental RNA percentages *p *and 100, respectively, and *a*_*i *_and *b*_*i *_are the intercept and slope of the  ratios versus the input placental RNA fraction for probe *i. *For the population of observed fractions at each input placental RNA fraction, the mean and median were calculated, along with the 2.5, 25, 75, and 97.5 percentile boundaries (Figure 1).

For endogenous transcript abundance estimation experiments, linear regression analysis was performed on seven yeast spike-in probe mean normalized log_10_[intensity] values for each microarray and the results were used to back-calculate estimated copy numbers for endogenous transcripts as



where *C*_*hmi *_is the microarray-estimated number of copies per hybridization for probe *i*, *I*_*i *_is the normalized log_10_[intensity] for probe *i*, and *a *and *b *are the intercept and slope of spike-in control probe microarray signal intensities versus. input spike-in transcript copy numbers. Dividing these values by the estimated number of cells represented in each hybridization,



converts them to estimates of transcript copies per cell. Amounts of total RNA extracted per cell for the four tissue types (EM 3.0 pg/cell, PL 2.0 pg/cell, ES 2.3 pg/cell, TS 3.0 pg/cell) were estimated from cell counts, RNA yields, and in the case of E12.5 embryo and placenta, our estimate that the average cell volume in these tissues is approximately 1.5 × 10^-9 ^cm^3 ^per cell (data not shown).

For measurement of abundances of mouse endogenous gene and spiked-in yeast transcripts in total RNA and labeled/amplified target mixtures by qPCR, linear regression of threshold cycle (*C*_*t*_) values versus input spike-in transcript copy numbers in a standard was used to back-calculate copy numbers per well of the transcripts in the total RNA samples and labeled/amplified target mixtures. These results were converted to copies per cell as follows:



In the case of endogenous mouse transcript measurements, results from both the microarray and qPCR were normalized to *Gapd *expression.

All microarray data will be deposited to the public repositories Gene Expression Omnibus at NCBI [30,31] and ArrayExpress at EBI [32,33] as soon as possible.

## Additional data files

The following additional data are available with the online version of this paper. Additional data file 1 is a table containing a standardized naming scheme for NIA oligonucleotide microarray platforms. Additional data file 2 is a table containing additional information on previous NIA microarray platforms and how they relate to that presented in this work. Additional data file 3 contains annotation of all probes in the NIA 44K Mouse Microarray v2.1.

## Supplementary Material

Additional File 1 A standardized naming scheme for NIA oligonucleotide microarray platforms. Click here for file

Additional File 2 Additional information on previous NIA microarray platforms and how they relate to that presented in this work. Click here for file

Additional File 3 Annotation of all probes in the NIA 44K Mouse Microarray v2.1 Click here for file

## Figures and Tables

**Table 1:** Yeast controls used in this study with corresponding qPCR primers

Yeast intronic/intergenic control transcript	Vector name ATCC number GenBank Accession	Insert size (bp)	Copies spiked/5 μg total RNA	Forward/reverse qPCR oligo sequence	Optimal concentration	Amplicon		Intron spanned?
							
						Size	T_m_	
YPL075W_16_412249_415357_INTRON_9_759	pNIAysic-1 MBA-201 DQ023287	630	1.00E+04	5'-CCTACTTGATAAAGCCACATACCTCTACCTCTTCTATTAG-3' 5'-TTGCGTTACTCTATTAATAATCCATAGTTGGAAC-3'	300 nM 50 nM	134 bp	73.4°C	No
YPL081W_16_404945_406039_INTRON_8_508	pNIAysic-2 MBA-202 DQ023288	400	1.00E+05	5'-CGACACTTCAGGTAAAGCGTTCCGAAGTAATTCAAC-3' 5'-TCTCAAACCTAACACATTTCTGTATTAAGCCTAG-3'	300 nM 300 nM	129 bp	75.8°C	No
NOT:D_1493031-1494574_553-1543	pNIAysic-3 MBA-203 DQ023289	997	1.00E+06	5'-TTACCATTCACTCCATGATGTCGTACCTGTTACACTAC-3' 5'-CGGTACATGTTATTACCAGAAAAAGATGTATATCC-3'	300 nM 300 nM	145 bp	79.8°C	No
YER133W_5_432491_433954_INTRON_178_702	pNIAysic-4 MBA-204 DQ023290	428	1.00E+07	5'-GTCGAGATAGCCGAGATAATGTGTGTG-3' 5'-GCAAGGGGGATTTTTCTGAATATGG-3'	300 nM 300 nM	136 bp	76.5°C	No
YNL162W_14_331319_332151_INTRON_5_516	pNIAysic-5 MBA-205 DQ023291	367	1.00E+08	5'-TGCAGCAACAGAGTATCATATGCATGG-3' 5'-CACTGCACAATCTGAAGATAGCGAGG-3'	300 nM 300 nM	145 bp	77.7°C	No
YNL302C_14_62942_61957_INTRON_21_571	pNIAysic-6 MBA-206 DQ023292	416	1.00E+09	5'-ATTTCCCATTACCTGATAAATTGAAGTTCATC-3' 5'-TTTGTATAGTTGGCTCAAAATATTCTCTCCAC-3'	900 nM 300 nM	100 bp	73.8°C	No
YBL087C_2_60732_59815_INTRON_43_546	pNIAysic-7 MBA-207 DQ023293	436	1.00E+010	5'-GCAGATGAAGTGATACCTGTCAATATTCATG-3' 5'-AGAAATAACATTTCGATGGTTATCCATTAGTATG-3'	300 nM 300 nM	128 bp	76.2°C	No

**Table 2:** Yeast controls with corresponding *in situ*-synthesized 60-mer oligonucleotide probes

Control transcript	NIA probe ID	60-mer oligonucleotide microarray probe sequence
NIA yeast control 1	Z10000036-1	5'-TTCAAGGGACAAATAACAGGATAAAACGTAATGTCAGGACACAAAGTGTGCCATCAACTT-3'
NIA yeast control 2	Z10000039-1	5'-TCTTCATAGAATACTTTTTTTTTCGGAGAAAACCTTTACACTGAACTCCCGACACTTCAG-3'
NIA yeast control 3	Z10000041-1	5'-TTTAATTATTCTTATTTCGCTTTTTTTCTCAAGGTGACCTGTTGTATCACGTTAGCTGAA-3'
NIA yeast control 4	Z10000020-1	5'-TCATCCGGCCGGCGCCTCCCATATTCAGAAAAATCCCCCTTGCTCACACTAAAAAAAGAA-3'
NIA yeast control 5	Z10000021-1	5'-TCAGATTGTGCAGTGATATTCTTTGAGGAAGGAAACGTAGAGGGGATAAGTTGGATAACT-3'
NIA yeast control 6	Z10000026-1	5'-CATTTACCGAACGAATGAGTTAAACTATTATGATATAATTGCTGTAATTGTGGAGAGAAT-3'
NIA yeast control 7	Z10000002-1	5'-AAAGTAAAGTTCCAAGATTTCATTTTGCTGGGTACAACAGAATTAAACAGAGGTTTAAAA-3'

**Table 3:** qPCR primer pairs used to quantitate endogenous transcripts in this study

Gene symbol	Forward/reverse qPCR oligo sequence	Optimal concentration	Amplicon		Intron spanned?
				
			Size	T_m_	
*Ank*	5'-AGTACCATAGTACACTCGGTTACCTGTCCTG-3'	900 nM	114 bp	78.8°C	Yes
	5'-GCAAAGCTTTAAGTCGTAATCTAGCATCC-3'	50 nM			
					
*Axl/Ufo*	5'-CGACTACCTGCGTCAAGGAAATCG-3'	300 nM	112 bp	82.8°C	Yes
	5'-AAAACTTGGCCGGTCTCGAGG-3'	300 nM			
					
*Cd34*	5'-TGCTCTGGAATCCGAGAAGTGAGG-3'	300 nM	140 bp	78.0°C	Yes
	5'-TCAGCCTCAGCCTCCTCCTTTTC-3'	300 nM			
					
*Dnchc1*	5'-AACTAAACCCAGCCATTCGGCC-3'	300 nM	98 bp	84.3°C	No
	5'-TTGCGTTGGCGGGTGACAG-3'	900 nM			
					
*Gap43*	5'-GAGAAGGGAAGGAGAGAAGGCAGG-3'	900 nM	131 bp	79.5°C	Yes
	5'-TCCGGCTTGACACCATCTTGTTC-3'	900 nM			
					
*Gapd*	5'-CGGAGTCAACGGATTTGGTCGTAT-3'	900 nM	214 bp	82.6°C	Yes
	5'-GAAGATGGTGATGGGCTTCC-3'	300 nM			
					
*H19*	5'-AGCTAACACTTCTCTGCTGCTCTCTGG-3'	300 nM	144 bp	81.4°C	Yes
	5'-ATCTTCTTGATTCAGAACGAGACGGAC-3'	900 nM			
					
*Hand1*	5'-GAGATGTATACCTGAGAGCAACAGGCATGATAGGTAG-3'	300 nM	113 bp	75.1°C	No
	5'-CTTCTCCTTCATTTCTTTCCTTTTCCTTC-3'	900 nM			
					
*Hif1a*	5'-GTCAGCAGTACATGGTAGCCACAATTG-3'	900 nM	139 bp	74.4°C	No
	5'-GATCCAGGCTTAACAATTCCATAGGC-3'	300 nM			
					
*Hmga1*	5'-AATTCAGGAGGATGAACATCTGACGC-3'	900 nM	114 bp	77.3°C	No
	5'-TCTGTTCACAAACTACCTCTGGACGG-3'	50 nM			
					
*Hprt1*	5'-AACAATGCAAACTTTGCTTTCCCTG-3'	300 nM	123 bp	80.1°C	Yes
	5'-TCAAATCCAACAAAGTCTGGCCTG-3'	300 nM			
					
*Igf2bp3*	5'-AAGTATACATTCTCACAGAGACAGGATCGAGTGACTG-3'	900 nM	126 bp	81.5°C	No
	5'-AAAGACAGATTTGCTTAACCAACAGACG-3'	900 nM			
					
*Lpl*	5'-TTTCCAGCCAGGATGCAACATTG-3'	300 nM	105 bp	82.3°C	No
	5'-TGAATGGAGCGCTCATGCGAG-3'	900 nM			
					
*Myo1b*	5'-AATACACACCTTGTACCAATCAGCTCTCTC-3'	900 nM	143 bp	76.1°C	No
	5'-TGATAAGAAGAGGCTGAGAGCCGTTC-3'	900 nM			

**Table 4:** Expressed genes and transcripts in developing mouse tissues and cultured stem cells

	EM	PL	ES	TS	Any tissue	All tissues
mRNAs/cell	851,087	519,688	400,045	568,196		
Features ≥ 1 CpC	13,718	10,559	9,667	9,840	14,908	8,073
U-clusters ≥ 1 CpC	11,360	8,828	8,101	8,271	12,264	6,838
Transcripts ≥ 1 CpC	11,762	9,108	8,357	8,534	12,739	7,037
Mean copies per cell	1.09	0.63	0.51	0.56		
Median copies per cell	0.79	0.45	0.36	0.40		

U-clusters and transcripts from the NIA mouse gene index were considered expressed if microarray features measured absolute expression estimated at one copy per cell or more. Copy-number estimates from expressed transcripts were summed to estimate the number of mRNA molecules per cell for each tissue, as well as the mean and median copy numbers. Microarray features corresponding to expressed genes and transcripts were mapped to the NIA Gene Index to calculate the number of U-clusters (loci) and transcripts expressed in each tissue.

**Table 5:** Pairwise comparison of expressed transcript sets in developing mouse tissues and cultured cells

Total expressed features	Overlapping features	EM	PL	ES	TS
13,718	EM		9,840	9,212	9,314
10,559	PL			8,508	8,881
9,667	ES				8,816
9,840	TS				
					
Total expressed U-clusters	Overlapping U-clusters	EM	PL	ES	TS

11,360	EM		8,271	7,749	7,853
8,828	PL			7,181	7,492
8,101	ES				7,435
8,271	TS				
					
Total expressed transcripts	Overlapping transcripts	EM	PL	ES	TS

11,762	EM		8,516	7,980	8,090
9,108	PL			7,386	7,718
8,357	ES				7,657
8,534	TS				

Sets of microarray features measuring expressed genes (≥ 1 copy per cell) were compared pairwise to calculate the number of members common to each pair. By matching microarray features to the NIA Gene Index, numbers of U-clusters (loci) and transcripts expressed in common were derived for each pairwise comparison. Signal intensities which were lower than those for all spike-in controls, as well as saturated signals, were not converted to copy number estimates (see Materials and methods), so these calculations may underestimate the number of expressed genes.

## References

[B1] Ko MS (1990). An 'equalized cDNA library' by the reassociation of short double-stranded cDNAs.. Nucleic Acids Res.

[B2] Waterston RH, Lindblad-Toh K, Birney E, Rogers J, Abril JF, Agarwal P, Agarwala R, Ainscough R, Alexandersson M, An P (2002). Initial sequencing and comparative analysis of the mouse genome.. Nature.

[B3] Schadt EE, Edwards SW, GuhaThakurta D, Holder D, Ying L, Svetnik V, Leonardson A, Hart KW, Russell A, Li G (2004). A comprehensive transcript index of the human genome generated using microarrays and computational approaches.. Genome Biol.

[B4] Singh-Gasson S, Green RD, Yue Y, Nelson C, Blattner F, Sussman MR, Cerrina F (1999). Maskless fabrication of light-directed oligonucleotide microarrays using a digital micromirror array.. Nat Biotechnol.

[B5] Hughes TR, Mao M, Jones AR, Burchard J, Marton MJ, Shannon KW, Lefkowitz SM, Ziman M, Schelter JM, Meyer MR (2001). Expression profiling using microarrays fabricated by an ink-jet oligonucleotide synthesizer.. Nat Biotechnol.

[B6] Dudley AM, Aach J, Steffen MA, Church GM (2002). Measuring absolute expression with microarrays with a calibrated reference sample and an extended signal intensity range.. Proc Natl Acad Sci USA.

[B7] van Bakel H, Holstege FC (2004). In control: systematic assessment of microarray performance.. EMBO Rep.

[B8] Ashburner M, Ball CA, Blake JA, Botstein D, Butler H, Cherry JM, Davis AP, Dolinski K, Dwight SS, Eppig JT (2000). Gene ontology: tool for the unification of biology. The Gene Ontology Consortium.. Nat Genet.

[B9] Carter MG, Hamatani T, Sharov AA, Carmack CE, Qian Y, Aiba K, Ko NT, Dudekula DB, Brzoska PM, Hwang SS, Ko MS (2003). In situ-synthesized novel microarray optimized for mouse stem cell and early developmental expression profiling.. Genome Res.

[B10] Sharov AA, Piao Y, Matoba R, Dudekula DB, Qian Y, VanBuren V, Falco G, Martin PR, Stagg CA, Bassey UC (2003). Transcriptome analysis of mouse stem cells and early embryos.. PLoS Biol.

[B11] Carter MG, Piao Y, Dudekula DB, Qian Y, VanBuren V, Sharov AA, Tanaka TS, Martin PR, Bassey UC, Stagg CA (2003). The NIA cDNA project in mouse stem cells and early embryos.. C R Biol.

[B12] Zhang W, Morris QD, Chang R, Shai O, Bakowski MA, Mitsakakis N, Mohammad N, Robinson MD, Zirngibl R, Somogyi E (2004). The functional landscape of mouse gene expression.. J Biol.

[B13] Parrish ML, Wei N, Duenwald S, Tokiwa GY, Wang Y, Holder D, Dai H, Zhang X, Wright C, Hodor P (2004). A microarray platform comparison for neuroscience applications.. J Neurosci Methods.

[B14] Grate L, Ares M (2002). Searching yeast intron data at Ares lab web site.. Methods Enzymol.

[B15] Ares Lab Yeast Intron Database. http://www.cse.ucsc.edu/compbio/yeast_introns/currentDB/stuff.html.

[B16] Tanaka S, Kunath T, Hadjantonakis AK, Nagy A, Rossant J (1998). Promotion of trophoblast stem cell proliferation by FGF4.. Science.

[B17] Van Ness J, Hahn WE (1980). Sequence complexity of cDNA transcribed from a diverse mRNA population.. Nucleic Acids Res.

[B18] Axel R, Feigelson P, Schutz G (1976). Analysis of the complexity and diversity of mRNA from chicken liver and oviduct.. Cell.

[B19] Holland MJ (2002). Transcript abundance in yeast varies over six orders of magnitude.. J Biol Chem.

[B20] Hastie ND, Bishop JO (1976). The expression of three abundance classes of messenger RNA in mouse tissues.. Cell.

[B21] Bishop JO, Morton JG, Rosbash M, Richardson M (1974). Three abundance classes in HeLa cell messenger RNA.. Nature.

[B22] Jongeneel CV, Iseli C, Stevenson BJ, Riggins GJ, Lal A, Mackay A, Harris RA, O'Hare MJ, Neville AM, Simpson AJ, Strausberg RL (2003). Comprehensive sampling of gene expression in human cell lines with massively parallel signature sequencing.. Proc Natl Acad Sci USA.

[B23] Hubner K, Fuhrmann G, Christenson LK, Kehler J, Reinbold R, De La Fuente R, Wood J, Strauss JF, Boiani M, Scholer HR (2003). Derivation of oocytes from mouse embryonic stem cells.. Science.

[B24] NIA Mouse Gene Index 2.0. http://lgsun.grc.nia.nih.gov/geneindex1/index.html.

[B25] Pertea G, Huang X, Liang F, Antonescu V, Sultana R, Karamycheva S, Lee Y, White J, Cheung F, Parvizi B (2003). TIGR Gene Indices clustering tools (TGICL): a software system for fast clustering of large EST datasets.. Bioinformatics.

[B26] Kent WJ (2002). BLAT - the BLAST-like alignment tool.. Genome Res.

[B27] Eyras E, Caccamo M, Curwen V, Clamp M (2004). ESTGenes: alternative splicing from ESTs in Ensembl.. Genome Res.

[B28] Bozdech Z, Zhu J, Joachimiak MP, Cohen FE, Pulliam B, DeRisi JL (2003). Expression profiling of the schizont and trophozoite stages of *Plasmodium falciparum *with a long-oligonucleotide microarray.. Genome Biol.

[B29] NIA Array Analysis Tool. http://lgsun.grc.nia.nih.gov/ANOVA/index.html.

[B30] Gene Expression Omnibus. http://www.ncbi.nlm.nih.gov/geo.

[B31] Edgar R, Domrachev M, Lash AE (2002). Gene Expression Omnibus: NCBI gene expression and hybridization array data repository.. Nucleic Acids Res.

[B32] ArrayExpress. http://www.ebi.ac.uk/arrayexpress.

[B33] Brazma A, Parkinson H, Sarkans U, Shojatalab M, Vilo J, Abeygunawardena N, Holloway E, Kapushesky M, Kemmeren P, Lara GG (2003). ArrayExpress - a public repository for microarray gene expression data at the EBI.. Nucleic Acids Res.

