# Supplementary material for: Transcript copy number estimation using a mouse whole-genome oligonucleotide microarray
Source: Genome Biol. 2005 Jun 30;6(7):R61. doi: 10.1186/gb-2005-6-7-r61 (PMC1175992; doi:10.1186/gb-2005-6-7-r61)
Supplement: Additional File 1 — A standardized naming scheme for NIA oligonucleotide microarray platforms. [file gb-2005-6-7-r61-S1.doc]

| NIA standardized name[[1]](#footnote-2) | Main features and alias | Gene content | Format | AMADID[[2]](#footnote-3) | Initial pubs. |
| --- | --- | --- | --- | --- | --- |
| NIA Mouse 15K Microarray v1.0 (cDNA clones on membrane) |  | NIA 15K cDNA clones | cDNA membrane | - | Tanaka{Tanaka, 2000 #11}; Kargul{Kargul, 2001 #13} |
| NIA Mouse 7.4K Microarray v1.0 (cDNA clones on membrane) |  | NIA 7.4K cDNA clones | cDNA membrane | - | VanBuren{VanBuren, 2002 #6} |
| NIA Mouse 22K Microarray v1.0 (Development 60-mer Oligo) | Prototype 22K  NIA 22K Mouse (Development) v1.0 | NIA 15K cDNA clones NIA 7.4K cDNA clones | 22K 60-mer oligo | 11321 | Carter{Carter, 2003 #4} |
| NIA Mouse 22K Microarray v1.1 (Development 60-mer Oligo) | Commercial 22K  Agilent Mouse (Development) Microarray] | NIA 15K cDNA clones NIA 7.4K cDNA clones | 22K 60-mer oligo | 11472 | Carter{Carter, 2003 #4} |
| NIA Mouse 22K Microarray v2.0 (Development 60-mer Oligo) | Revised/Improved 22K  NIA 22K Mouse (Developmental) v2.0 | NIA 15K cDNA clones NIA 7.4K cDNA clones ”named genes” | 22K 60-mer oligo | 12165 | this report |
| NIA Mouse 44K Microarray v1.0 (Development-Toxicology 60-mer Oligo) | 44K format testing  NIA DEV+NIEHS TOX 44K | NIA Mouse Gene Index v2.0 NIEHS Mouse 22K Toxicogenomics (redundancy-depleted) | 44K 60-mer oligo | 12201 | this report |
| NIA Mouse 44K Microarray v2.0 (Whole Genome 60-mer Oligo) | Prototype 44K  Mouse 44K v1.0 | NIA Mouse Gene Index v2.0 yeast spike-in controls | 44K 60-mer oligo | 12463 | this report |
| NIA Mouse 44K Microarray v2.1 (Whole Genome 60-mer Oligo) | Commercial 44K | NIA Mouse Gene Index v2.0 yeast spike-in controls | 44K 60-mer oligo | 12799 | this report |

**Supplemental Table 1: NIA mouse microarray platforms**

In an effort to clarify the origin and content of the various microarray platforms developed at NIA, we propose the standard names listed above, to be used in future references to the various designs.

1. DNA microarrays produced according to these NIA designs are available commercially from Agilent Technologies. However, The National Institutes on Health and The National Institute on Aging do not endorse these products or make any claims or guarantees as to their quality or performance. Names shown here are based on NIA designs, and manufacturers’ products may use different nomenclature. [↑](#footnote-ref-2)
2. The AMADID is Agilent’s unique identifier for microarray designs which will enable other investigators to order slides manufactured according to NIA designs. [↑](#footnote-ref-3)
