# Supplementary material for: Transcript copy number estimation using a mouse whole-genome oligonucleotide microarray
Source: Genome Biol. 2005 Jun 30;6(7):R61. doi: 10.1186/gb-2005-6-7-r61 (PMC1175992; doi:10.1186/gb-2005-6-7-r61)
Supplement: Additional File 2 — Additional information on previous NIA microarray platforms and how they relate to that presented in this work. [file gb-2005-6-7-r61-S2.doc]

|  | NIA standardized name[[1]](#footnote-2) | unique probes | spike-in probes | probe origin | identified genes/ | Identified transcripts | unidentified ESTs |
| --- | --- | --- | --- | --- | --- | --- | --- |
| a | NIA Mouse 22K Microarray v1.0 (Development 60-mer Oligo) | 21,910 | - | 21,910 new | 15,101 | 15,913 | 4,587 |
| b | NIA Mouse 22K Microarray v1.1 (Development 60-mer Oligo) | 20,280 | - | 20,280 from a | 14,826 | 15,594 | 3,370 |
| c | NIEHS Toxicogenomics Microarray | 20,865 | - | - | 17,644 | 18,172 | 2,215 |
| d | NIA Mouse 22K Microarray v2.0 (Development 60-mer Oligo) | 21,044 | - | 6,759 from a 3,268 from c 11,017 new | 18,845 | 19,134 | 1,625 |
| e | NIA Mouse 44K Microarray v1.0 (Development-Toxicology 60-mer Oligo) | 38,508 | - | 6,798 from a 20,693 from c 11,017 from d | 24,747 | 27,056 | 3,705 |
| f | NIA Mouse 44K Microarray v2.0 (Whole Genome 60-mer Oligo) | 42,354 | 63 | 6,709 from a 9,468 from c 11,015 from d 15,099 new | 29,801 | 32,132 | 5,803 |
| g | NIA Mouse 44K Microarray v2.1 (Whole Genome 60-mer Oligo) | 41,041 | 7 | 6,507 from a 9,323 from c 10,338 from d 14,180 from f 686 new[[2]](#footnote-3) | 29,597 | 31,932 | 4,733 |

**Supplemental Table 2: NIA mouse microarray design history**

Each revision/expansion of the NIA 60-mer oligonucleotide mouse microarray has incorporated probes from preceding designs with newly-designed probes, while reducing redundancy and increasing gene/transcript coverage.

1. DNA microarrays produced according to these NIA designs are available commercially from Agilent Technologies. However, The National Institutes on Health and The National Institute on Aging do not endorse these products or make any claims or guarantees as to their quality or performance. Names shown here are based on NIA designs, and manufacturers’ products may use different nomenclature. [↑](#footnote-ref-2)
2. In NIA Mouse 44K Microarray v2.0, 3,327 oligos from previous designs did not match the target gene’s major transcript with the longest ORF, so a second oligo for the major transcript of these genes was added. [↑](#footnote-ref-3)
